# Obesity prevention in early life: an opportunity to better support the role of Maternal and Child Health Nurses in Australia

**DOI:** 10.1186/s12912-015-0077-7

**Published:** 2015-05-08

**Authors:** R. Laws, K. J. Campbell, P. van der Pligt, K. Ball, J. Lynch, G. Russell, R. Taylor, E. Denney-Wilson

**Affiliations:** Centre for Physical Activity and Nutrition Research, Deakin University, 221 Burwood Highway, Burwood, VIC 3125 Australia; School of Population Health, University of Adelaide, Adelaide, Australia; Faculty of Health, University of Technology, Sydney, Australia; University of Otago, Dunedin, New Zealand; Centre for Obesity Management and Prevention Research Excellence in Primary Health Care (COMPaRE-PHC), ᅟ, Australia

**Keywords:** Obesity prevention, Children, Infant feeding, Primary health care, Families, Nurses

## Abstract

**Background:**

Because parents with young children access primary health care services frequently, a key opportunity arises for Maternal and Child Health (MCH) nurses to actively work with families to support healthy infant feeding practices and lifestyle behaviours. However, little is known regarding the extent to which MCH nurses promote obesity prevention practices and how such practices could be better supported.

**Methods:**

This mixed methods study involved a survey of 56 MCH nurses (response rate 84.8 %), 16 of whom participated in semi-structured qualitative interviews. Both components aimed to examine the extent to which nurses addressed healthy infant feeding practices, healthy eating, active play and limiting sedentary behavior during routine consultations with young children 0–5 years. Key factors influencing such practices and how they could be best supported were also investigated. All data were collected from September to December 2013. Survey data were analysed descriptively and triangulated with qualitative interview findings, the analysis of which was guided by grounded theory principles.

**Results:**

Although nurses reported measuring height/length and weight in most consultations, almost one quarter (22.2 %) reported never/rarely using growth charts to identify infants or children at risk of overweight or obesity. This reflected a reluctance to raise the issue of weight with parents and a lack of confidence in how to address it. The majority of nurses reported providing advice on aspects of infant feeding relevant to obesity prevention at most consultations, with around a third (37 %) routinely provided advice on formula preparation. Less than half of nurses routinely promoted active play and only 30 % discussed limiting sedentary behaviour such as TV viewing. Concerns about parental receptiveness and maintaining rapport were key barriers to more effective implementation.

**Conclusion:**

While MCH nurses are well placed to address obesity prevention in early life, there is currently a missed public health opportunity. Improving nurse skills in behaviour change counseling will be key to increasing their confidence in raising sensitive lifestyle issues with parents to better integrate obesity prevention practices into normal MCH service delivery.

**Electronic supplementary material:**

The online version of this article (doi:10.1186/s12912-015-0077-7) contains supplementary material, which is available to authorized users.

## Background

Childhood obesity remains one the most important public health challenges globally, potentially affecting 60 million children by 2020 [1]. Increasingly, children are becoming overweight at a relatively young age, which affects metabolic health, both in childhood and later in life [2]. In 2011–2012, 17.8 % of Australian children aged 2–4 years were overweight and 5.0 % obese. In the USA, over a quarter (26.7 %) of children aged two-five years were overweight and 12.1 % obese in 2009–10 [3]. Globally, the prevalence of overweight and obesity amongst preshoolers (aged three-five years) has increased dramatically since the 1990s, confirming the need for effective interventions commencing in early life [1].

Several obesity risk factors may be important at this life stage. Early infant feeding practices (formula feeding, feeding to a schedule) influences weight gain in infancy [4, 5] and rapid infant weight gain is one of the strongest risk factors for obesity later in life [6]. Early introduction of solids (before four months of age) [7], poor diet quality [8] low levels of physical activity [9] and electronic media use [8] such as television viewing and other screen time have also been implicated. Children’s weight status, food intakes and food preferences track over time [10, 11] and parents play a primary role in shaping these behaviours through different feeding styles and the food and physical activity environments they provide [12]. Together these findings suggest that early childhood is a critical time in which many risk factors for overweight and obesity emerge, and when habits are formed, providing an opportunity for establishing and promoting behaviours that will affect weight gain and health across the life course.

Because parents access Primary Health Care (PHC) so frequently, an unparalleled opportunity exists to incorporate obesity prevention initiatives, which could reach most of the population [13]. On average parents make 11 visits to general practitioners and 14 visits to Maternal and child health (MCH) nurses in the first year of their child’s life. Because the majority of these visits are unrelated to illness [14], this provides a key opportunity for intervention at a time when parents are potentially more receptive to healthy living advice. The Maternal and Child Health Service in Victoria, Australia is a universal free service provided by MCH Nurses to all families of children from birth to six years. The service consists of ten key age and stage consultations including a home visit shortly after birth, and then consultations at two, four and eight weeks; four, eight, twelve and 18 months; and two and three and a half years of age [15]. The aim of these consultations is to support parents in the areas of maternal health, parenting and child health and development. A key component of the service is monitoring of child growth and development along with linking parents to community programs and referral to other health professionals [15]. MCH nurses in Victoria are registered nurses with qualifications in midwifery and child and family health.

However, little is known about the obesity prevention practices of MCH nurses in Australia or their equivalent counterparts in other countries. The limited number of existing studies to date have been small scale qualitative studies exploring how nurses talk to parents about their children’s weight, [16] or their perceived role in obesity prevention and any barriers to interacting with parents [17–19]. We are unaware of any previous studies that have used a mixed methods approach to more comprehensively describe how MCH nurses incorporate obesity prevention practices in their routine consultations with families and what factors might influence such practices. Given the high frequency with which parents have contact with MCH nurses and their role in growth monitoring and child development, more insight is needed into nurses’ current obesity prevention practices and the scope to influence such practices.

Therefore the aims of this paper were to: 1) examine the child obesity prevention practices of MCH nurses, 2) explore the key factors influencing such practices and 3) identify opportunities to enhance and support MCH nurses in this role.

## Methods

This study used a mixed methods design [20] involving the collection of quantitative survey data followed by qualitative interviews with survey participants to further elaborate and expand on survey responses. The survey aimed to examine the child obesity prevention practices of MCH nurses (study aim one) and the interviews aim to explore the factors influencing such practices and how they could be best supported (study aim two and three). The methods were mixed at the point of data collection (individual were asked to elaborate and explain their survey responses), during analysis (qualitative data was explored to help explain survey findings) and in the final phase of interpretation (quantitative and qualitative data were triangulated).

### Sample

MCH nurses from two of 31 Local Government Areas (LGAs) in Melbourne, Australia were invited to participate in this study. The LGAs were selected on the basis of their willingness to participate in a larger feasibility study of an intervention (Growing healthy, www.growinghealthy.org.au) for promoting healthy infant feeding practices amongst socioeconomically disadvantaged parents. As such the LGAs selected had a high proportion of disadvantaged communities, defined on the basis of an area wide indicator (socioeconomic index for areas [21]). One LGA is located in the outer eastern and northeastern suburbs of Melbourne, with a population of 144,541 [22], with 18 MCH nurses available to participate in the study. The other LGA is located in the outer south-eastern suburbs of Melbourne and is Victoria’s most populous and fastest growing municipality, with a 2011 census population of 252,382 [23], with 48 nurses available to participate in the study.

MCH nurses in each of these LGAs were briefed about the purpose of the study and invited to participate in the survey by email. The email was sent by MCH managers on behalf of the research team and this provided a link to the survey. Three subsequent reminders were sent, one week apart. Nurses were asked in the survey if they were interested in participating in a telephone interview to further discuss this issue with a member of the research team. All those expressing interest in an interview were subsequently emailed to organise a suitable interview time. All nurses completing the survey were offered the opportunity to enter a prize draw for a $100 retail gift voucher, no monetary compensation was provided for participating in an interview. The study was approved by Deakin University Human Research Ethics Committee. Completion of the survey was taken as participants providing informed consent, and verbal consent was obtained and recorded prior to participation in the interviews.

### Data collection tools

#### Survey

The survey (Additional file 1) was developed based on the literature and informed by previous theoretical models developed and applied to the management of lifestyle risk factors in PHC [24, 25]. The 24 question survey took around 15 to 20 min to complete. It included questions on current MCH nurse behaviours, including:

- the number and type of consultations currently conducted with young children
- the frequency with which MCH nurses undertook various activities related to the promotion of healthy eating and active play in young children in a typical consultation (five point Likert scale anchored by never (0 % of consultations) to most of the time (>75 % of consultations),
- Perceived confidence in undertaking activities described in (b), (five point Likert scale anchored by ‘not confident at all’ and ‘extremely confident,’)
- Views and knowledge regarding infant feeding and TV watching in young children (four point Likert scale anchored by ‘strongly disagree’ and ‘strongly agree’)
- Current use of government guidelines to inform nurse practice on infant feeding, healthy eating, active play and sedentary behaviour
- Perceived ease of access to relevant support materials and referral services
- Perceived barriers to promoting healthy weight gain (rating a list of predefined barriers on a three point Likert scale anchored by ‘not a barrier at all’ and ‘substantial barrier’)
- Training received in the past two years on a predefined list of topics related to obesity prevention as well as future training needs (open response).

The survey tool was pilot tested prior to the study to assess face validity and ease of comprehension with two MCH nurses not involved in the study. Minor amendments to the wording of questions were made in response to this feedback.

### Interview guide

An initial interview guide was developed to explore the survey responses in more detail. In particular, factors influencing the obesity prevention practices of MCH nurses, and opportunities to support such practices were expanded upon. The interview guide covered the following topics:

- The current focus of the MCH nurses’ role
- Approaches used by the MCH nurses to monitor infant/child growth and identify young children at risk of overweight/obesity
- Perceptions of barriers and facilitators that influence whether MCH nurses discuss healthy eating, active play and limiting screen time with parents
- Perceptions of parental receptiveness to advice and perceived effectiveness of discussions with parents about healthy eating, active play and limiting screen time
- Perceptions of current government guidelines on key behaviours including breastfeeding initiation and duration, introduction of solids and limiting screen time
- Nurses’ ideas regarding ways to support a MCH role in promoting healthy eating, active play and limiting screen time

The interviews took around 30 min to complete but ranged from 20 to 60 min duration. Interviews were conducted over the phone by RL and Pv and tape recorded with the participant’s permission.

### Data analysis

#### Survey analysis

The survey data were analysed descriptively using SPSS 22.0. The frequency of practices related to obesity prevention were collapsed into three categories: 1) never and rarely (0-25 % of consultations), 2) sometimes and often (26-75 % of consultations), or 3) most of the time (>75 % of consultations). Perceived confidence related to these practices was collapsed into three categories: 1) not confident at all, 2) somewhat confident or 3) very or extremely confident. Attitudinal and knowledge items were collapsed into two categories: 1) strongly disagree and disagree; and 2) strongly agree and agree. Open responses on requests for future training were coded by a single researcher into common categories.

### Interview analysis

**Fig. 1:**
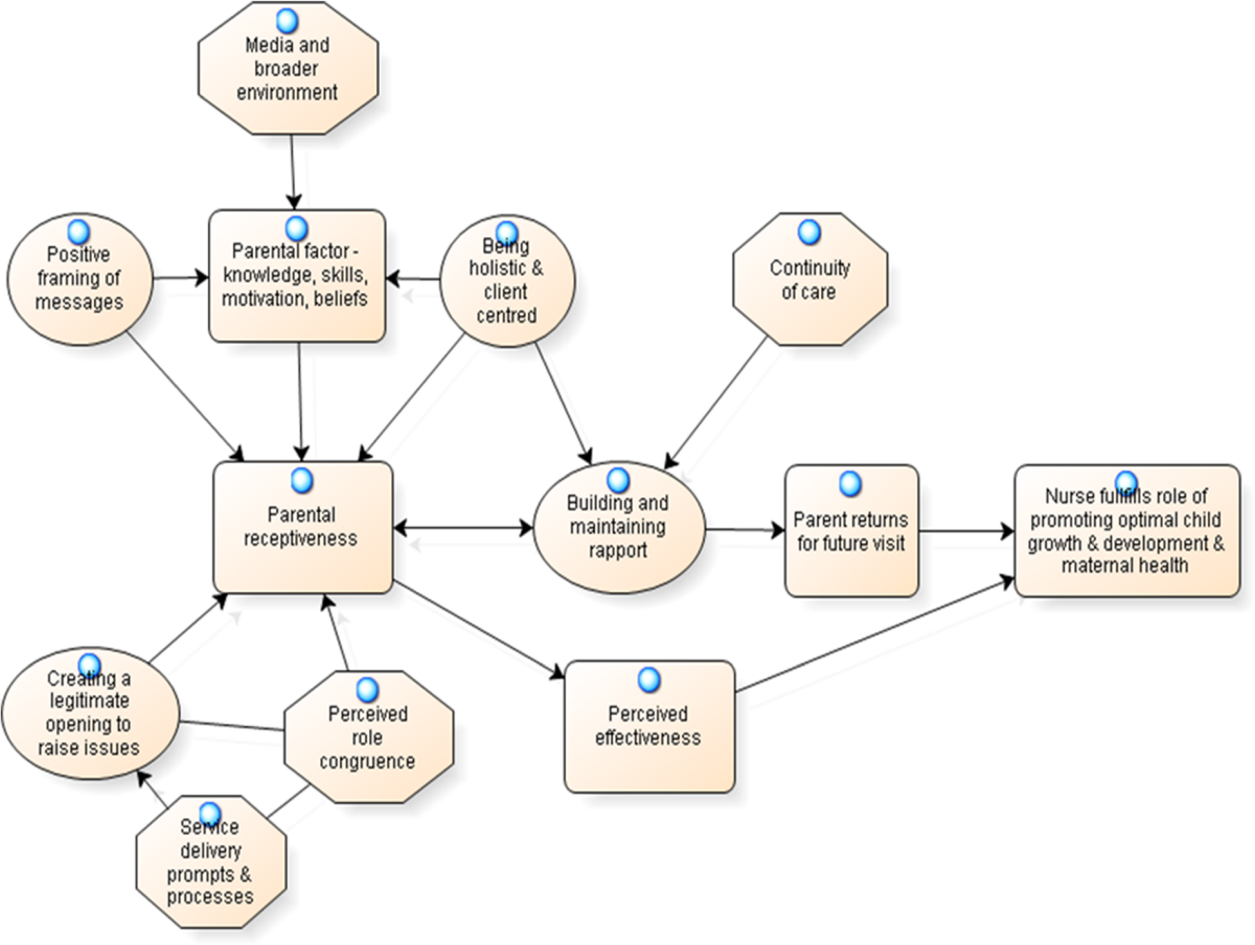
Integrated framework of key factors influencing MCH nurse obesity prevention practices

All interviews were transcribed verbatim and imported into Nvivo 9 which was used for coding, sorting and retrieval of data. The analysis process involved the following steps: 1) checking the accuracy of transcripts against audio recording during which notes were made by the first author (RL) on key issues arising and any tensions in the data (for example opposing views); 2) coding of the data into broad categories using an inductive approach guided by the research aims; 3) further focused coding within broad categories to identify common and divergent views. In line with grounded theory principles [26], this included paying attention to the conditions that gave rise to an issue, the context in which the issue was embedded, the strategies used by nurses to manage the issue and context, and the consequences for nurse practices. In addition survey findings were used as a basis for further exploration of qualitative data to help illuminate the findings. For example, codes related to the reasons nurses stated for providing less advice on active play than on healthy eating were examined to help explain this quantitative finding. An integrated framework of factors influencing obesity prevention practices was developed throughout the analysis process by examining how codes were linked and related using a visual model (see Fig. 1). The final explanatory model (Fig. 1) developed during the coding process was confirmed by the re-reading of all interview transcripts when slight modifications and refinements to the model were made.

All coding was initially undertaken by the first author (RL). RL is a dietitian and researcher with extensive experience in working with PHC practitioners in the management of lifestyle risk factors. Data coded under each main category was reviewed by a second author (PvP) with any discrepancies in coding and interpretation resolved through discussion.

## Results

### Sample characteristics

A total of 56 out of 66 (84.8 %) MCH nurses across the two LGAs completed the quantitative survey, with 16 interviews completed from the 20 who initially expressed an interest in participating in an interview. The remaining four interviews were unable to be scheduled within the timeframe of the study. All participants were female, the majority were over 50 years of age and working part time, and there was a wide range of experience from 18 months to 34 years (Table 1). Interview participants were broadly representative of those completing the survey with the exception that a higher proportion of interview participants worked full time and had less than five years experience (Table 1).

**Table 1:** Characteristics of MCH nurses participating in survey and interviews

**Characteristics**	**Survey (n = 56)**	**Interview (n = 16)**
	**Number (%)**	**Number (%)**
Gender		
Female (n = 51)	51 (100)	16 (100)
Age (years) (n = 51)		
20-29	2 (3.9)	0 (0)
30-39	3 (5.9)	1 (6.3)
40-49	11 (21.6)	4 (25.0)
50-59	25 (49.0)	9 (56.3)
60+	10 (19.6)	2 (12.5)
Years working in profession (n = 51)		
0-5	15 (29.4)	8 (50.0)
6-10	7 (13.7)	1 (6.3)
11-15	14 (27.5)	3 (18.8)
16-20	3 (5.9)	0 (0)
20+	12 (23.5)	4 (25.0)
Working hours (n = 51)		
Full time	14 (27.5)	6 (37.5)
Part time	37 (72.5)	10 (62.5)

### Obesity prevention practices (Table 2)

**Table 2:** Frequency of MCH nurses undertaking the following as part of a typical consultation (n = 54) and perceived confidence (n = 53)

**Activity**	**Never/rarely (0-25 % consults)**	**Sometimes/often (26-75 % of consuls)**	**Most of the time (>75 % consults)**	**Very/Extremely confident**
Measure height and weight of children UNDER 2 years	2 (3.7)	6 (11.1)	46 (85.2)	53 (100.0)
Plot height and weight on a growth chart (for children under 2 years)	5 (9.3)	7 (12.9)	42 (77.8)	53 (100.0)
Measure height and weight for children OVER 2 years	6 (11.1)	6 (11.1)	42 (77.8)	53 (100.0)
Calculate body mass index (BMI) for children OVER 2 years and plot on a BMI percentile chart	12 (22.2)	6 (11.1)	36 (66.7)	41 (77.4)
Use growth or BMI charts to identify infants/children who are at risk of overweight or obesity	12 (22.2)	10 (18.5)	32 (59.3)	40 (75.5)
Provide advice or support to encourage continuation of breastfeeding in breastfeeding mothers	0 (0.0)	22 (40.7)	32 (59.3)	53 (100.0)
Provide advice on correct formula preparation to parents who are formula feeding their infants	12 (22.2)	22 (40.7)	20 (37.0)	53 (100.0)
Provide advice on sleep and settling techniques for infants	0 (0.0)	29 (53.7)	25 (46.3)	52 (98.1)
Provide advice on WHEN to introduce solid foods to infants	0 (0.0)	18 (33.3)	36 (66.7)	53 (100.0)
Provide advice on HOW to introduce solid foods to infants	0 (0)	20 (37.0)	34 (63.0)	53 (100.0)
Talk to parents about eating their meals with their infants/children	3 (5.6)	21 (38.9)	30 (55.6)	53 (100.0)
Talk to parents about limiting infants and young children’s intake of sweetened drinks (e.g. juice and soft drinks)	0 (0)	19 (35.2)	35 (64.8)	53 (100.0)
Talk to parents about offering water as the child’s main drink (after 12 months of age)	1 (1.9)	17 (31.5)	36 (66.7)	53 (100.0)
Talk to parents about limiting infants and young children’s TV viewing and other electronic media use (DVDs, computers etc.)	7 (13.0)	30 (55.6)	17 (30.4)	48 (90.6)
Talk to parents about increasing active play for infants and young children	8 (14.9)	22 (40.7)	24 (44.4)	51 (96.2)
Talk to parents about increasing their infants/children’s fruit and vegetable intake	4 (7.4)	20 (37.0.)	30 (55.6)	53 (100.0)
Talk to parents about limiting infants/children intake of foods high in fat/salt/sugar (e.g. cakes, biscuits, lollies, chips, take away foods etc.)	6 (11.1)	16 (29.6)	32 (59.3)	52 (98.1)

#### Growth monitoring and identifying ‘at risk’ infants/children

Although measurement of height/length and weight was commonplace, a significant portion of nurses (22.2 %) rarely used growth charts to identify infants or children at risk of overweight or obesity (Table 2). This is despite virtually all nurses reporting having easy access to standard growth charts (Table 3) and the majority being confident in using these charts to identify those at risk of overweight or obesity (Table 2). The qualitative interviews highlighted that many nurses felt less confident about broaching the topic of overweight with parents, due in part perhaps to a lack of ‘back up’ information to provide to parents if the child was overweight.

*“I want to look more at obesity… that’s an area that I thought I need to know a bit more about…as soon as you go down that path, parents put barriers up and you can feel them shut down because they just don’t want to hear that their child’s overweight” (nurse 13).*

Nurses also perceived that parents generally did not recognise their child to be overweight or they were not concerned about their child weight, considering a ‘chubby baby’ to be a healthy baby. However, some nurses used growth or BMI charts to legitimise raising the topic with parents:

*“…I discuss the graph with the parents. So if you've got a child sort of consistently…sitting on the fiftieth percentile and yet weight's over ninety-eighth percentile it's easy for parents to see why it’s a concern. I think by discussing the graph and showing it it’s in their face, they can’t ignore it” (nurse 12*).

Nurses who tended not to use the charts felt that they were not well understood by parents or indeed the nurses themselves and the charts had a range of limitations:

*“I don’t look at them (growth or BMI charts) all that much because it doesn’t take into effect their race, the parents’ size or anything like that” (nurse 2).*

**Table 3:** Access to support materials and use of guidelines

**Access to Support Materials (n = 56)**	**Number (%)**
Standard growth charts for infants 0–2 years	56 (100.0)
BMI percentile charts for children 2–8 years	52 (92.9)
Dietitian to refer families with young children 0–5 year for nutrition advice	43 (76.8)
Lactation consultant to refer mothers to for breastfeeding advice	54 (96.4)
Educational materials on infant feeding	52 (92.9)
Education materials on healthy eating for toddlers and pre-schoolers	52 (92.9)
Education materials on promoting active play in young children	27 (48.2)
Education materials on limiting sedentary behaviour	15 (26.8)
None of the above	1 (1.8)
**Use of Guidelines (n = 52)**	
Sedentary behaviour in young children 0–5 years	15 (28.8)
Infant feeding	49 (94.2)
Healthy eating in young children (0–5 years)	50 (96.2)
Physical activity in young children (0–5 years)	31 (59.6)
Don’t use any published guidelines	2 (3.8)

Interestingly, almost a third of nurses surveyed agreed that it was easy to identify overweight infants and young children just by looking at them, and around one quarter erroneously believed that accelerated weight gain in infancy is not related to the development of overweight in childhood (Table 4).

**Table 4:** MCH nurse views and knowledge on infant feeding and TV watching in young children (0–5 years) (n = 53)

	**Strongly disagree/disagree No (%)**	**Strongly agree/agree No (%)**
**Growth monitoring and identification of at risk children**		
Accelerated weight gain in infancy is NOT related to the development of overweight in childhood	40 (75.5)	13 (24.5)
It is easy to identify overweight infants and young children just by looking at them	37 (69.8)	16 (30.2)
It is easy to identify infants and young children who are at risk of becoming overweight	25 (47.2)	28 (52.8)
**Parental feeding styles**		
A good way to get infants and young children to eat healthy food is to offer a food as a reward (e.g. offering dessert if they eat all their vegetables)	47 (88.7)	6 (11.3)
Parents should offer an alternative food if their infant/child doesn’t eat the food offered	48 (90.6)	5 (9.4)
Parents should encourage their infant/child to eat all the food on their plate	47 (88.7)	6 (11.3)
If a parent continues to offer foods their infant hasn’t previously enjoyed, they will come to enjoy them	10 (18.8)	43 (81.1)
An infant knows when s/he is full	16 (30.2)	37 (69.8)
**Timing on introduction of solids**		
An infant under 6 months sometimes needs more than breastmilk or formula to be full	27 (50.9)	26 (49.1)
**TV and small screen use**		
TV is educational for children under 2 years of age	45 (84.9)	8 (15.1)
Children under 2 should NOT be allowed to watch TV	34 (64.2)	19 (35.8)
The recommendation to limit TV viewing and the use of other electronic media (DVDs, computers etc.) to less than one hour per day for children 2–5 years is unrealistic for most parents	26 (49.1)	27 (50.9)

#### Provision of advice and support on infant feeding

The majority of nurses surveyed reported providing advice on aspects of infant feeding at most consultations, including a high proportion routinely providing advice and support on breastfeeding (59.3 %), when (66.7 %) and how (63 %) to introduce solids, limiting sweetened drinks (60 %), promoting water as the main drink after 12 months of age (66.7 %), limiting intake of high fat/salt/sugar foods (55 %) and sharing family meals (52 %) (Table 2). However, a minority were routinely providing advice on correct formula preparation (37.0 %) (Table 2). Overall confidence in providing advice and support on all these topics, access to education materials on infant feeding and healthy eating for children and access to health professionals (e.g. dietitian and lactation consultant) for referral purposes were all very high (Tables 2 and 3).

Qualitative interviews revealed that addressing infant feeding was considered central to the MCH role and a key priority in promoting child growth and development, as discussed by this nurse:

*It is a priority, definitely…it needs to be mentioned any time when the parent comes in because you and I know that’s going to affect their child’s development. So it’s really important for them to be getting the right sort of nutrients (nurse 11).*

### Perceptions of infant feeding guidelines – Introduction of solids

Despite current recommendations suggesting that parents should wait until infants are around 6 months of age before introducing solids, almost half of nurses surveyed agreed or strongly agreed with the statement that “ an infant under six months sometimes need more than breastmilk or formula to be full”.

The qualitative interviews highlighted that although nurses were supportive of the 6 month guideline, there was widespread recognition that advice should be tailored to the needs of the baby.

*…we do see a lot of kids from four to five months starting to show that great interest in food and wanting to eat…So we have to sort of treat every child as an individual…So we can’t say six months 100 % for every child (nurse 9).*

This tailored approach stems in part from conflicting recommendations around timing of introduction of solids:

*…there’s a proponent that it (introducing solids) is between four to six months so it reduces the incidence of allergy etc., then on the other hand there’s the other side of the story they say no don’t introduce too early, you have child obesity problems…but it all depends on what it is for some mums and what their experience has been as well… I guess I’m a bit of both either way (nurse 6).*

### Parental feeding practices

The survey data indicated that most nurses demonstrated a good understanding of appropriate parental feeding practices to promote healthy growth including not using food as a reward and not pressuring infants/children to finish a meal, managing food rejection by offering repeated exposures and supporting infant self regulation of appetite (Table 4). However, when asked about the type of advice nurses provided on infant feeding during the interviews, few nurses specifically mentioned advising parents about these parental feeding practices. The focus was on what to feed rather than how to feed.

#### Promotion of active play

In contrast to the strong focus on infant feeding, just over 44 % of nurses reported discussing active play with parents in most consultations, despite almost all nurses feeling very or extremely confident to do so (Table 2). Just under half reported having access to educational materials on promoting active play in young children and 60 % used guidelines on active play to inform their practice (Table 3). Nurses provided several reasons for the relative lack of focus on active play:

- diet and infant feeding were perceived to be more important in influencing growth and development than active play
- a perceived higher degree of parental interest in infant feeding compared to active play
- active play is perceived by nurses as less important for younger babies because they are not moving as much as older children
- the existence of fewer service delivery prompts, such as active play not being part of compulsory topics on a computer program used by MCH nurses and being less frequently mentioned in parent education materials.

*“I think it is a lot to do with parents being interested in it and also measuring their growth, I suppose you associate a lot with what they’re eating and drinking. So you don’t really so much associate that with the play, although of course it does impact on it. So I suppose that’s the bigger focus, it seems to be” (nurse 4).*

In contrast, other nurses recounted focusing on active play right from birth as part of discussions with parents about day time routines for their infants such as a. ‘feed, play and sleep’ routine. Others discussed active play and limiting TV in the context of encouraging parents to interact with their infants to promote learning and development.

*“…right from birth, talking about tummy time and getting the baby on the floor, getting active down on the floor with the baby, keeping the baby safe too, you know, when they’re older trying to limit television, reading to their child, doing more involved interactive things than just being babysat by the television” (nurse 12).*

### Advice on limiting screen time

Only 30 % of those surveyed reported routinely (in most consultations) talking to parents about limiting infants’ and children’s screen time such as watching TV and use of other electronic media, despite most feeling confident to do so (Table 2). Only just over a quarter of nurses reported having access to educational materials on limiting sedentary behaviour and less than a third (28.8 %) used guidelines to inform their practice in this area (Table 3).

In the qualitative interviews nurses generally recognised this was an area they needed to emphasise more, but many felt reluctant or uncomfortable suggesting limiting screen time to parents as this was considered ‘overstepping’ the mark:

*I feel that it’s something that you say to families and they don’t take on,… because it maybe sounds a little bit like you’re trying to control too many things that go on at home,… except I think more and more we’re seeing the research is showing it does need to be limited. But it almost sounds a little …like an old person giving advice, like the old grandma saying you’ll get square eyes. But it is important, it is definitely important and needs to be said. So I just need to get past that (nurse 4).*

A few nurses also mentioned that discussing screen time with some parents was not a priority because these parents had more pressing issues to contend with (e.g. family violence) and time available in the consultation was short. Other nurses were discouraged by the negative reactions received by parents about limiting screen time and the fact that they believed that their advice was unlikely to be received well:

*And I do say it is recommended by the experts…that kids don’t have screens before the age of two, and the parents just sort of gawk at you and they just go “Really? What am I supposed to do with them then?” It’s kind of like, “I don’t know, interact with them maybe, read them a book, go to the park, go for a walk”, you know, just sort of give them alternatives. Again, you can see them going “Yeah, right”. (nurse 16)*

Only just over a third of nurses surveyed (36 %), agreed or strongly agreed with the recommendation that children under two should not be allowed to watch TV. Slightly more (51 %) agreed or strongly agreed with limiting screen time in two to five year olds to less than one hour per day (Table 3). This was in line with the qualitative data, with all nurses interviewed agreeing that screen time recommendations were unrealistic for most parents. Nurses discussed the view that screens are an embedded part of our culture, a reality of life, used by parents for their own entertainment and as a babysitter.

Despite this, just under half of nurses interviewed (n = 7) discussed framing advice around limiting screen time in a way that was more acceptable to parents. This included:

- highlighting that screens can be too stimulating for babies and should be avoided to promote better sleep and settling;
- acknowledging that TV and screen time is a reality and rather than having no screen time aiming to limit screen time and not have the TV on all day;
- encouraging parents to choose age appropriate TV programs/screen time and watch it together so it is more interactive/social

Two nurses specifically mentioned not raising the issue of excess screen time at all; rather they focused on encouraging active play, which they considered to be a more positive approach.

### Key barriers to addressing obesity prevention in routine practice

From the survey data, parent related barriers were rated by MCH nurses as the main obstacles to addressing obesity prevention in routine practice, with almost two thirds of nurses considering parent’s lack of recognition of child overweight, and negative responses to having child weight raised in the consultation as substantial barriers (Table 5). In contrast, service related barriers were considered less important barriers, with 29 % considering a lack of referral pathways to provide additional/ongoing support to parents a substantial barrier and a minority (24 %) rating lack of time a substantial barrier. Practitioner barriers related to the perceived effectiveness of their advice, role congruence and their own knowledge, confidence or own lifestyle habits were considered the least important barriers (Table 5).

**Table 5:** Key barriers to addressing obesity prevention in routine practice (n = 55)

**Barrier**	**Not a barrier at all No (%)**	**Minor barrier No (%)**	**Substantial barrier No (%)**
Parent related barriers			
Parents don’t recognise their child is overweight (n = 51)	5 (9.8)	13 (25.5)	33 (64.7)
Some parents react negatively to me raising the issue of their child’s weigh (n = 51)	9 (17.6)	8 (15.7)	34 (66.7)
Parent is not motivated to change the diet or lifestyle of the family (n = 51)	4 (7.8)	21 (41.2)	26 (51.0)
Socio-economic factors affect the ability of families to make a change (e.g. cost of healthy food) (n = 51)	7 (13.7)	23 (41.1)	21 (41.2)
Addressing the child’s weight is not a priority for parents (n = 50)	10 (20.0)	21 (42.0)	19 (38.0)
A lack of relevance to the parent or child’s presenting issue (n = 51)	15 (29.4)	26 (51.0)	15 (29.4)
Service related barriers			
A lack of referral pathways to provide additional/ongoing support for parents if required (n = 51)	13 (25.5)	23 (45.1)	15 (29.4)
A lack of time (n = 51)	20 (39.2)	19 (33.9)	12 (23.5)
A lack of appropriate education materials for parents available in my clinic/practice (n = 51)	11 (21.6)	30 (58.8)	10 (19.6)
A lack of support from managers/supervisors for me to undertake this work in my role (n = 51)	44 (86.3)	6 (11.8)	1 (2.0)
Practitioner related barriers			
My advice and support does little to promote the adoption of a healthy lifestyle for parents and their children (n = 51)	15 (29.4)	28 (54.9)	8 (15.7)
I feel uncomfortable raising the issue of infants and young children’s weight with parents (n = 51)	32 (62.7)	16 (31.4)	3 (5.9)
Lack of relevance to my role (n = 51)	37 (72.5)	12 (23.5)	2 (3.9)
I lack knowledge about how to most effectively prevent child overweight and obesity. (n = 51)	42 (82.4)	7 (13.7)	2 (3.9)
I lack confidence to counsel parents about healthy eating and physical activity (n = 51)	43 (84.3)	8 (15.7)	0 (0)
My own lifestyle habits (n = 51)	46 (90.2)	5 (9.8)	0 (0)
I don’t find this kind of work professionally rewarding n = 51	49 (96.1)	2 (3.9)	0 (0)

In line with this, the majority of nurses (n = 11) in qualitative interviews revealed that they perceived of a lack of parental receptiveness to advice on healthy eating or active play to be the key barrier to promoting healthy weight gain. Low levels of parental receptiveness were considered to reflect:

- Parents lacking knowledge and skills, particularly related to healthy eating and cooking
- Parents’ own unhealthy lifestyle behaviours and lack of desire to change
- Cultural, family and peer influence

Nurses frequently raised the importance of cultural influences on perceptions relevant to child weight, e.g. cultural beliefs regarding infant feeding (timing, choices, quantities) and perceptions that big babies are healthier. Not surprisingly, grandparents, who for many families provide childcare, were reported to be influential in reinforcing cultural values. The influence of grandparents was not just an issue amongst specific cultural groups, but a key factor influencing parental receptiveness to nurse advice amongst many families.

*Some specific cultural groups they say that a big. fat baby is a healthy baby …In this day and age a lot of parents have gone back to work and they are cared for by grandparents. So grandparents have a big part to play as well. I have had families that come in and say my mother said that my child is underweight and then when you plot them on the graph their…weight is higher than the height…they perceive that is underweight. It’s the grandparents (nurse 6)*

Only a minority of nurses interviewed (n = 6) discussed service related barriers, with lack of time the most frequent of these barriers if there were other pressing concerns to be addressed. The only barrier arising in the qualitative interviews that was not reflected in the survey results was a lack of continuity of care with parents. This was considered to be a barrier to building rapport which made the provision of tailored advice and support more difficult.

### Supporting obesity prevention practices

#### Continuing professional development

Most nurses reported receiving training in the past two years on infant feeding topics such as breastfeeding and the introduction of solids, with the majority also receiving training on healthy eating for infants and toddlers (Table 6). Only 28 % reported receiving training in the past two years on active play, and few (n = 5) had received recent training on limiting sedentary behaviour or overweight and obesity management in children. The most frequently requested areas for future training included healthy eating and active play for young children as well as limiting sedentary behaviour, overweight and obesity prevention and management and behaviour change techniques (Table 6).

**Table 6:** Previous training and request for future training

**Topics**	**Received training in past 2 years (n = 51)**	**Request for future training (n = 51)**
	**No (%)**	
Breastfeeding (e.g. benefits, support, techniques etc.)	40 (78.4)	3 (5.9)
Introduction of solids to infants (e.g. timing, types of foods etc.)	40 (78.4)	4 (7.8)
Healthy infant feeding practices (e.g. eating together as a family not using food as a reward)	27 (52.9)	9 (17.6)
Healthy eating for young children	30 (58.8)	18 (35.3)
Active play for young children	14 (27.5)	18 (35.3)
Limiting sedentary behaviour	5 (9.8)	13 (25.5)
Overweight/obesity management in children	5 (9.8)	13 (25.5)
Overweight/obesity prevention in children	10 (19.6)	13 (25.5)
Behaviour change techniques	10 (19.6)	12 (23.5)
No training in past 2 years	2 (3.9)	n/a

Qualitative interviews provided further insights into nurses’ desires to learn. For healthy eating, nurses wanted to know more about how to tailor advice to different cultures, portion sizes for infants and children of different ages, as well as key nutrient requirements, specifically requirements for calcium and iron. For active play, nurses wanted more active play ideas at different ages as well as groups and programs operating in the community they could recommend to parents. In terms of limiting sedentary behaviour, nurses wanted more information on the basis for such recommendations and how messages about limiting screen time could be framed positively for parents. For obesity prevention and management, nurses wanted further insight into the role of genetics versus the environment in influencing weight gain in early life as well how to sensitively broach the topic of overweight so not to offend parents. Related to this, several nurses mentioned wanting to gain more skills in promoting behaviour change:

*“ it’s one thing to have some knowledge and it’s a totally different thing to actually apply it in your life. It’s something about not just informing or giving knowledge but how do you actually do it in ways that people will then integrate it?” (nurse 15).*

#### Access to support materials and services

In line with the survey data about access to support materials (Table 3), most of the nurses who were interviewed discussed wanting more educational materials to give to parents on active play and sedentary behaviour. Nurses also discussed that it would be useful to have more visual information to give to parents on healthy eating including pictures of portion sizes, textures and finger food ideas.

Also reflecting the survey data, most nurses who were interviewed felt they had reasonably good access to health professionals and programs to refer parents to for additional support. Waiting times, particular for lactation services, was the main limitation identified.

#### Service and organisational factors

In the interviews, nurses identified a number of service and organisational factors that could be implemented to support obesity prevention practices. These included better role modelling within the service, for example, not having televisions in waiting rooms, increasing the length of consultations and legitimising nurses role in obesity prevention. While nurses discussed that their role in growth monitoring was well accepted, the emphasis and parental expectation was on ensuring adequate weight gain in infancy rather than preventing overweight.

*I think we need to be given the legitimate okay to talk about it..At least if we are given the okay to do that and then the parents themselves realise that oh yes this is a space that we’ll be talking about..At the moment it’s (weight) just recorded, it says on the computer and very briefly mentioned and shown. .. See the thing is, with the parents we’re also trying to strike a balance between - initially the weight gain for the baby to grow, grow, grow and then boom they’re overweight….they’re (parents) very, very concerned about weight gain…whereas for us we do need to…look at the total picture…So I think…legitimising and giving us the okay, the permission, to talk about the baby’s weight and height and BMI in the relationship to child obesity. I think we need to revisit that (nurse 6).*

### Integrated framework of factors influencing obesity prevention practices

A central theme emerging from the analysis of the qualitative interviews was the importance of parental receptiveness and maintaining rapport with parents in influencing obesity prevention practices (Fig. 1).

*“some people, they’re here, they talk to you, they’re easy to engage, others, you can just know that there are barriers there and they don’t really want to know what you’ve got to say…So there are times when we’d rather keep them coming back and build a relationship, so we don’t go there and push against any of their barriers in an effort to keep them coming back” (nurse 13).*

Maintaining rapport was considered critical in encouraging parents to attend for future visits, allowing nurses to fulfil their role in monitoring and supporting child growth and development. As shown in Fig. 1, nurses used a number of strategies to promote parental receptiveness. Firstly, being ‘holistic and client centred’ by tailoring the consultation to the needs of the parents or the baby was considered important in promoting parental receptiveness. This included prioritising parental concerns, taking into account parent’s knowledge, beliefs and motivations and the influence of the wider family and peers. Prioritising parental concerns meant that on occasions there was insufficient time in the consultation to address healthy eating or active play, but nurses rationalised that by maintaining good rapport, parents would return and these issues could be addressed in future consultations. Secondly, nurses aimed to promote parental receptiveness through the use of positive messages and reinforcement, for example, focusing on ‘what to do’ rather than ‘what not to do,’ while respecting parental choices. Thirdly, nurses used service delivery prompts and processes (such as parent education materials) to create a legitimate opening to discuss some issues that were considered more distal to their role, such as promotion of active play and limiting sedentary behaviour. Finally, a lack of continuity of care was considered a threat to both parental receptiveness and building rapport.

## Discussion

This paper is one of the first to explore, using a mixed methods approach, the obesity prevention practices of MCH nurses and the factors influencing them. Our findings suggest that MCH nurses frequently undertake growth monitoring and address infant feeding practices important in the prevention of obesity. MCH nurses are less likely, however, to use growth charts to identify children at risk of overweight and obesity, or to promote active play or provide advice about limiting sedentary behaviour. Qualitative analysis revealed that parental receptiveness and maintaining rapport with parents was a key driver of MCH nurse practices related to obesity prevention in infants and children. Solutions to improving obesity prevention practices may include further developing nurse counselling and behaviour change skills to enable them to raise sensitive issues in a way that engages rather than offends parents and aligning obesity prevention advice with nurses’ roles in promoting optimal growth and development.

Despite growth monitoring being a central role of MCH nurses, our findings suggest that there is room for improvement in using growth charts to identify infants and children at risk of overweight and obesity. A recent meta-analysis showed a consistent positive association between infant weight gain in the first year of life and subsequent childhood and adult obesity [27]. The same study also demonstrated a risk score combining birthweight and infant weight gain together with mother’s body index and sex may allow early stratification of infants at risk of childhood obesity.

Under-utilization of charts by pediatric health care providers [28] has predominately been attributed to the inability of parents to recognise overweight in their offspring and the perception that parents will react negatively to practitioners raising the issue of their child’s weight [16, 17, 19, 29]. Both practitioners [16, 19] and parents [30] have reported that growth charts can be useful in objectively raising weight related concerns, as long as the terminology used is acceptable. Parents prefer terms such as ‘heavy for their age’ ‘large’ ‘gaining weight too rapidly’ to the terms overweight or obese which are considered by parents to have negative connotations and be associated with stigma and prejudice in society [30]. As such, nurse training should focus on further developing counselling and behaviour change skills such as reflective listening, motivational interviewing and goal setting to assist nurses in raising the issue of weight in a sensitive and non-judgemental manner. A recent trial of the ‘Healthy Conversations” training program for health and social care practitioners in the UK has shown that a relatively short course focusing on skills to support behaviour change (using open discovery questions, listening, reflecting and goal-setting) was effective in changing practice over a one year period [31].

Overall MCH nurses in the study reported high rates of confidence and frequent provision of advice and support to parents on some aspects of infant feeding, which they considered central to their role such as breastfeeding and the basics of introducing solids. We also observed however that there was scope to improve provision of advice and support around other aspects of infant feeding including best practice formula feeding and parental feeding behaviours shown to be important in preventing obesity such as feeding to appetite, repeated neutral exposure to foods and not using food as a reward. The use of service delivery prompts such as including these topics in computer system and parent education materials may remind nurses to cover these issues as part of overall discussions regarding infant feeding.

To our knowledge, our study is the first to report on the extent to which MCH nurses address active play and limiting sedentary behaviour with young children. Both the survey and qualitative interviews suggested that MCH nurses were less likely to address promotion of these behaviours compared to addressing infant feeding topics. Nurses considered these topics to be less central to their role and they felt reluctant to discuss limiting screen time with parents because of concerns that these recommendations were unrealistic for parents. To address this gap in practice our findings indicate that it is important to provide MCH nurses with further training on the guidelines for active play and limiting sedentary behaviour, with information on practical tips for parents, and ways of communicating positive messages to parents, particularly related to limiting screen time. Nurses may also benefit from further guidance about how active play and limiting sedentary behaviour can be aligned with key ages and stages of child development, for example, limiting TV in babies to avoid overstimulation and promote settling. Incorporating these topics into existing parent education materials will provide a prompt and legitimate opening for nurses to raise these topics. Most importantly, increasing nurse confidence in behaviour change counselling may help to overcome their concerns about offending parents and lead to more productive and effective parent nurse relationship.

In line with previous research [16, 17, 19, 29] our qualitative findings highlight that parental receptiveness and maintaining rapport with parents is a key concern of MCH nurses and pivotal in influencing practices related to obesity prevention. Our integrated framework of factors influencing obesity prevention practices suggested that strategies to improve practice will need to 1) use service delivery prompts such as BMI chart/parent education materials to assist nurses in creating a legitimate opening to raise sensitive issues such as weight and screen time, 2) align advice on healthy eating, active play and screen time with child growth and development which is considered central to the nurse role, 3) positively frame obesity prevention messages such as screen time limits to promote parental receptiveness, 4) promote continuity of care with parents, and 5) increase nurse skills in behaviour change counselling so they are more confident to approach parents about sensitive topics without fearing this will impact negatively on the nurse-parent relationship.

This study has a number of limitations. It is based on a relatively small sample size of MCH nurses from two local government areas who agreed to take part based on their interest in participating in a child obesity prevention intervention and thus may have more interest and engagement in child obesity prevention than nurses in other areas. However, the response rate to the survey was high (85 %) and while nurses participating in the interviews were self-selected, they were broadly representative of those completing the survey. The survey tool has not been previously validated against other measures of practice (e.g. record review) and hence relies on self-report which is subject to over reporting of desirable clinical practices. Reliability testing of the survey has also not been conducted. The survey was however pilot tested for face validity and ease of comprehension of items prior to the study. A strength of the study was the triangulation of quantitative and qualitative data to further understand and explore child obesity prevention practices which helps to overcome some of the limitations of using survey data alone.

## Conclusion

Promoting healthy weight gain fits well with the MCH nurse role, and nurses routinely undertake growth monitoring and the provision of infant feeding advice relevant to obesity prevention. A major public health opportunity remains however in assisting nurses to more effectively promote aspects of infant feeding including best practice formula feeding and parental feeding behaviours as well as promoting active play and limiting sedentary behaviour. Supporting MCH nurses to embrace this opportunity must address concern about negative reactions from parents. Because parental receptiveness and maintaining rapport with parents is the key driver of nurse obesity prevention practices, improving behaviour change counselling skills will be critical to nurses raising weight related concerns with parents and addressing sensitive topics such as limiting screen time. At the service level, the use of tools such as BMI charts and integrating topics such as parental feeding behaviours, active play and screen time into parent education materials will help create legitimate openings to raise such topics with parents. With this additional support, MCH nurses are well placed to play an important role in obesity prevention in early life.

## Additional file

Additional file 1:
**MCH Nurse survey.**

## Abbreviations

MCH: Maternal and child health
PHC: Primary health care
