# Supplementary material for: Obesity prevention in early life: an opportunity to better support the role of Maternal and Child Health Nurses in Australia
Source: BMC Nurs. 2015 May 8;14:26. doi: 10.1186/s12912-015-0077-7 (PMC4429503; doi:10.1186/s12912-015-0077-7)
Supplement: Additional file 1: — MCH Nurse survey. [file 12912_2015_77_MOESM1_ESM.pdf]

Healthy Eating Lifestyle and Parenting (HELP) Project

Survey Introduction

This short 10-15 minute survey aims to examine the knowledge, attitudes and practices and support needs of primary health care practitioners on promoting healthy eating and active play in young children (0-5 years). Information from the survey will be used to inform the development of a training and support program for practitioners as part of the Healthy Eating, Lifestyle and Parenting (HELP) project. Your participation is greatly appreciated.

Your responses will be anonymous and confidential. All responses will be de-identified and your individual results will not be reported. If you need to stop before completing the survey your responses will be saved and you will be able to resume the survey where you left off.

Participants will have the opportunity to enter a prize draw for a $100 retail gift voucher.

Copy of page:

1. Approximately how many consultations would you have in a typical week with:

Infants and toddlers (0-2 years): __________

Young children (3-5 years): __________

2. Approximately, what proportion of the consultations with children (0-5 years), are for the following reason?

	None	A few (1-25%)	Some (26-50%)	Majority (51-75%)	Almost all (>75%)
a. Routine baby or child health checks	○	○	○	○	○
b. Immunisations	○	○	○	○	○
c. Breastfeeding advice or support	○	○	○	○	○
d. Other infant/child feeding advice or support (excluding breastfeeding)	○	○	○	○	○
e. Acute health problem	○	○	○	○	○
f. Chronic health problem	○	○	○	○	○
g. Other	○	○	○	○	○

Others (please specify)

____

3. Do you have easy access to the following? Please tick all that apply.

- ☐ Standard growth charts for infants 0-2 years
- ☐ BMI percentile chart for children aged 2-18 years
- ☐ Dietitian to refer families with young children 0-5 years for nutrition advice
- ☐ Lactation consultant to refer mothers to for breastfeeding advice
- ☐ Education materials for parents on infant feeding
- ☐ Education materials for parents on healthy eating for toddlers and pre-schoolers
- ☐ Education materials for parents on promoting active play in young children
- ☐ Education materials for parents on limiting sedentary activities (eg TV watching)
- ☐ None of the above

4. The following questions ask about how often you undertake various activities relating to the promotion of healthy eating and active play in young children (0-5 years).

As part of a typical consultation with parents of young children, about how often would you? Please tick one response for each statement.

	Never (0% of consultations)	Rarely (1-25% of consultations)	Sometimes (26-50% of consultations)	Often (51-75% of consultations)	Most of the time (>75% of consultations)
Measure height and weight of children UNDER 2 years	◯	◯	◯	◯	◯
Plot height and weight on a growth chart (for children under 2 years)	◯	◯	◯	◯	◯
Measure height and weight for children OVER 2 years	◯	◯	◯	◯	◯
Calculate body mass index (BMI) for children OVER 2 years and plot on a BMI percentile chart	◯	◯	◯	◯	◯
Use growth or BMI charts to identify infants/children who are at risk of overweight or obesity	◯	◯	◯	◯	◯
Provide advice or support to encourage continuation of breastfeeding in breastfeeding mothers	◯	◯	◯	◯	◯
Provide advice on correct formula preparation to parents who are formula feeding their infants	◯	◯	◯	◯	◯
Provide advice on sleep and settling techniques for infants	◯	◯	◯	◯	◯
Provide advice on WHEN to introduce solid foods to infants	◯	◯	◯	◯	◯
Provide advice on HOW to introduce solid foods to infants	◯	◯	◯	◯	◯
Talk to parents about eating their meals with their infants/children	◯	◯	◯	◯	◯
Talk to parents about limiting infants and young children’s intake of sweetened drinks (eg juice and soft drinks)	◯	◯	◯	◯	◯
Talk to parents about offering water as the child’s main drink (after 12 months of age)	◯	◯	◯	◯	◯
Talk to parents about limiting infants and young children's TV viewing and other electronic media use (DVDs, computers etc)	◯	◯	◯	◯	◯
Talk to parents about increasing active play for infants and young children	◯	◯	◯	◯	◯
Talk to parents about increasing their infants/children’s fruit and vegetable intake	◯	◯	◯	◯	◯
Talk to parents about limiting infants/children intake of foods high in fat/salt/sugar (eg cakes, biscuits, lollies, chips, take away foods etc)	◯	◯	◯	◯	◯
Refer parents to a website or smartphone app for information on infant feeding and/or active play	◯	◯	◯	◯	◯

5. The following statements ask about YOUR VIEWS on infant feeding and TV watching in young children (0-5 years).

Please indicate how much you agree or disagree with the statements by ticking one response for each statement.

	Strongly disagree	Disagree	Agree	Strongly agree
Accelerated weight gain in infancy is NOT related to the development of overweight in childhood	◌	◌	◌	◌
It is easy to identify overweight infants and young children just by looking at them	◌	◌	◌	◌
It is easy to identify infants and young children who are at risk of becoming overweight	◌	◌	◌	◌
A good way to get infants and young children to eat healthy food is to offer a food as a reward (e.g. offering dessert if they eat all their vegetables)	◌	◌	◌	◌
Parents should offer an alternative food if their infant/child doesn't eat the food offered	◌	◌	◌	◌
Parents should encourage their infant/child to eat all the food on their plate	◌	◌	◌	◌
If a parent continues to offer foods their infant hasn’t previously enjoyed, they will come to enjoy them	◌	◌	◌	◌
The best way to settle a crying infant is to feed him/her	◌	◌	◌	◌
Exclusive breastfeeding should be encouraged to all mothers for the first six months of their infant’s life	◌	◌	◌	◌
I believe I can influence a mother’s decision to exclusively breastfeed for the first six months of her infant’s life	◌	◌	◌	◌
An infant under 6 months sometimes needs more than breastmilk or formula to be full	◌	◌	◌	◌
An infant knows when s/he is full	◌	◌	◌	◌
TV is educational for children under 2 years of age	◌	◌	◌	◌
Children under 2 should NOT be allowed to watch TV	◌	◌	◌	◌
The recommendation to limit TV viewing and the use of other electronic media (DVDs, computers etc) to less than one hour per day for children 2-5 years is unrealistic for most parents	◌	◌	◌	◌
Some parents are influenced by information from websites or smartphone apps that is not accurate about infant feeding and/or active play	◌	◌	◌	◌
Websites and smartphone apps can be helpful for parents seeking information on infant feeding and/or active play	◌	◌	◌	◌
I would like to have reputable websites and smartphone apps to refer parents to for infant feeding and/or active play	◌	◌	◌	◌

6. How confident are you in undertaking the following? Please tick one response for each statement.

	Not at all confident	Somewhat confident	Very confident	Extremely confident
Measuring infants' height and weight and plotting on a growth chart	○	○	○	○
Calculating BMI for children 2 years and older and plotting on a BMI percentile chart	○	○	○	○
Identifying infants and young children who are at risk of overweight or obesity	○	○	○	○
Providing breastfeeding advice and support	○	○	○	○
Providing advice on correct formula preparation	○	○	○	○
Providing advice on sleeping and settling techniques for infants	○	○	○	○
Providing advice to parents regarding WHEN to introduce solid foods to infants	○	○	○	○
Providing advice to parents regarding HOW to introduce solid foods to infants	○	○	○	○
Talking to parents about eating their meals with their children	○	○	○	○
Talking to parents about limiting sugar sweetened drinks (eg juice and soft drinks)	○	○	○	○
Talking to parents about offering water as the child's main drink (after 12 months of age)	○	○	○	○
Talking to parents about limiting TV and other screen based activities	○	○	○	○
Talking to parents about increasing active play for infants and young children	○	○	○	○
Talking to parents about increasing their infant's/child's fruit and vegetable intake	○	○	○	○
Talking to parents about limiting infants/children intake of foods high in fat/salt/sugar (eg cakes, biscuits, lollies, chips, take away foods etc)	○	○	○	○
Referring parents to reputable websites or smartphone apps for infant feeding and/or active play	○	○	○	○

7. Do you use any published guidelines (e.g. from government or health bodies) to inform your practice and advice regarding the following? Please tick all that apply.

- ☐ Sedentary behaviour (e.g. TV watching) in young children (0-5 years)
- ☐ Infant feeding
- ☐ Healthy eating in young children (0-5 years)
- ☐ Physical activity in young children (0-5 years)
- ☐ None of the above

8. Please specify which guideline(s) you use.

9. Do you use any websites or smartphone apps to inform your practice and advice regarding the following? Please tick all that apply.

- ☐ Infant feeding
- ☐ Healthy eating in young children (0-5 years)
- ☐ Physical activity in young children (0-5 years)
- ☐ None of the above

10. Please specify which website(s) and app(s) you use.

11. To what extent are the following issues a barrier for you in talking to parents of young children (0-5 years) about healthy eating and active play?

	Substantial barrier	Minor barrier	Not a barrier at all
Parents don't recognise their child is overweight	○	○	○
Some parents react negatively to me raising the issue of their child's weight	○	○	○
Addressing the child's weight is not a priority for parents	○	○	○
Parent is not motivated to change the diet or lifestyle of the family	○	○	○
My advice and support does little to promote the adoption of a healthy lifestyle for parents and their children	○	○	○
Socio-economic factors affect the ability of families to make a change (e.g. cost of healthy food)	○	○	○
Lack of relevance to my role	○	○	○
A lack of relevance to the parent or child's presenting issue	○	○	○
A lack of support from managers/supervisors for me to undertake this work in my role	○	○	○
A lack of time	○	○	○
A lack of referral pathways to provide additional/ongoing support for parents if required	○	○	○
A lack of appropriate education materials for parents available in my clinic/practice	○	○	○
I lack confidence to counsel parents about healthy eating and physical activity	○	○	○
I lack knowledge about how to most effectively prevent child overweight and obesity.	○	○	○
I feel uncomfortable raising the issue of infants and young children's weight with parents	○	○	○
My own lifestyle habits	○	○	○
I don't find this kind of work professionally rewarding	○	○	○
Other	○	○	○

Other (please specify)

12. In the past 2 years have you had formal training (ie more than one hour of professional designed instruction) on any of the following (please tick all that apply):

- ☐ Breastfeeding (e.g benefits, support, techniques etc)
- ☐ Introduction of solids to infants (e.g. timing, types of foods)
- ☐ Healthy infant feeding practices (e.g. eating together as a family, not using food as a reward)
- ☐ Healthy eating for young children (0-5 years)
- ☐ Active play for young children (0-5 years)
- ☐ Limiting sedentary behavior (e.g. TV watching) in young children (0-5 years)
- ☐ Overweight /obesity management in children
- ☐ Overweight/ obesity prevention in children
- ☐ Behaviour change techniques
- ☐ None of the above

13. Would you be interested in additional training in the area of promoting healthy eating and active play in young children (0-5 years)?

- ○ Yes
- ○ No

14. Please specify the main topic areas you would like further training on:

15. What format would you like to receive additional training? Please tick all that apply.

- ☐ Workshop
- ☐ Self-study material (eg online module)
- ☐ Clinical supervision/mentoring

Other (please specify)

16. Are you...

- ○ Female
- ○ Male

17. What is your age?

- ○ 20-29 years
- ○ 30-39 years
- ○ 40-49 years
- ○ 50-59 years
- ○ 60+ years

18. What is your profession?

- ○ Maternal and Child Health Nurse
- ○ Midwife
- ○ Social Worker
- ○ Health Promotion Officer
- ○ Dietitian
- ○ Other (please specify)

19. How many years have you worked in this profession?

20. Do you work..

- ○ Full time
- ○ Part Time

If Part time, how many hours do you work each week, on average?

21. Would you like to be entered into the prize draw to win a $100 Coles Myer gift voucher?

- ○ Yes
- ○ No

22. The research team is in the process of developing a new program designed to promote healthy infant feeding practices and lifestyle behaviours for disadvantaged families. Would you be interested in hearing more about this program?

- ○ Yes
- ○ No

23. Would you be interested in participating in a short interview with a researcher to tell us more about your experience and views in promoting healthy eating and active play in infants and young children (this will take approximately 30 minutes)?

- Yes
- No

24. If you answered YES to any of the last 3 questions, please provide your details. This will be kept separately from your survey responses to ensure responses remain anonymous.

Name	
Day time contact number	
Email address	
Names of the main suburbs in which you work	
